# Thermo-sensitive ε-polylysine-heparin-poloxamer hydrogel-encapsulated BMSCs promote endometrial regeneration

**DOI:** 10.1016/j.mtbio.2025.101580

**Published:** 2025-02-15

**Authors:** Ruifang Han, Haiyi Zhou, Xingshan Liang, Siyi He, Xiaoming Sun, Yongge Guan, Yang Song

**Affiliations:** a School of Nursing, Guangzhou University of Chinese Medicine, Guangzhou, 510006, China; b The Third Affiliated Hospital of Guangzhou University of Chinese Medicine, Guangzhou, 510378, China

**Keywords:** Endometrial injury, Thermo-sensitive EPL-HP hydrogel-encapsulated BMSCs, Nrf2, SDF-1/CXCR4 axis

## Abstract

Endometrium plays a key role in embryo implantation and maintenance of pregnancy. However, to repair endometrial injury is still a challenge. In recent years, hydrogel materials have been widely used as effective support matrices to prevent intrauterine adhesions after endometrial injury. They can also be used as preparation scaffolds for encapsulating MSCs and certain therapeutic drugs. This study aimed to develop a preparation scaffold with high tissue affinity, high viscoelasticity and controlled release for repair of endometrial injury. The scaffold utilized heparin poloxamer (HP) as the matrix material and ε-polylysine (EPL) as the functional excipient to prepare a hydrogel that is suitable for endometrial adhesion and further encapsulate BMSCs. Furthermore, a strategy of the thermo-sensitive EPL-HP hydrogel-encapsulated BMSCs were used for better homing of BMSC after transplantation into the rat endometrial injury model, so as to exert the potential of endometrial regeneration by activating Nrf2 to regulate SDF-1/CXCR4 axis.

## Graphical abstract

**Figure:**
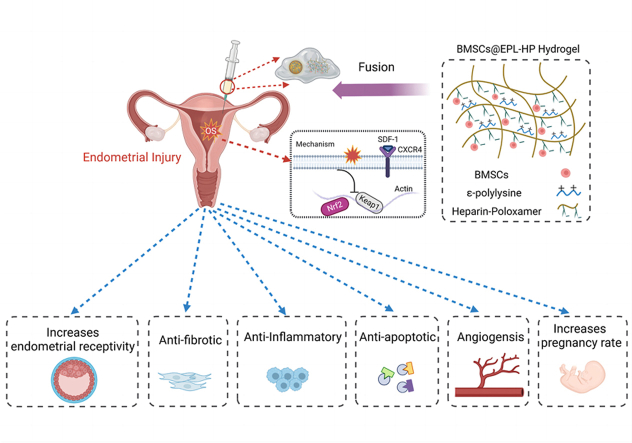

## 1 Introduction

Frequent intrauterine surgeries (such as myomectomy, removal of intrauterine foreign bodies after recurrent miscarriage, and induced abortion, etc.), infection, and ovulation-stimulating drugs could lead to damage to the endometrial basal layer, regenerative disorders of endometrial cells and glands, as well as damage to new blood vessels. As a result, endometrial regeneration is severely impaired, affecting reproductive health and causing female infertility, eventually posing a potential threat to population growth and social progress in the long run [1]. Thin endometrium and intrauterine adhesions are the results of poor regeneration following endometrial injury, leading to an incidence rate of infertility as high as 40 % [2], which has become a challenge for reproductologists. Currently, existing treatments cannot fundamentally solve the problem of endometrial regeneration.

Studies have shown that transplanting mesenchymal stem cells (MSCs) with self-renewal and multifunctional differentiation properties could promote endometrial regeneration, thereby improve the thickness and receptivity of the endometrium, and achieve the purpose of repairing damaged endometrium [3]. It is undoubtedly of important clinical application value. Compared with other MSCs, the application of bone marrow mesenchymal stem cells (BMSCs) has begun to shift from animal model research to clinical application [4]. However, existing research has found [5] that after transplanting MSCs through local in situ injection into the uterine cavity, the survival rate, homing rate, proliferation and differentiation rates of MSCs did not reach the ideal state. At present, researchers have found that using certain biological materials as scaffolds (such as polylactic-glycolic acid (PLGA) scaffolds [6], hydrogels [7] and nanostructured lipid carriers [8], etc.) to deliver MSCs could improve the survival rate of MSCs and thus improve the therapeutic effect.

As a scaffold material for cell transplantation, thermo-sensitive hydrogel could mediate cell adhesion, growth and deposition of extracellular matrix [9]. This characteristic ensures the survival of BMSCs in the scaffold materials. In addition, due to its capability of in situ gelation, the thermo-sensitive hydrogel could promote the spread of BMSCs to the injury site and playing a good blocking role [10,11]. Nowadays, thermo-sensitive hydrogels have been widely used in disease treatment, which improved wound healing through inflammation inhibition [12,13]. Moreover, XU et al. found [14,15] that thermo-sensitive ɛ-polylysine heparin poloxamer hydrogel (EPL-HP) with a suitable keratinocyte growth factor (KGF) release profile may be a more promising approach to repair the injured endometrium, which has a close relatively relationship between autophagy and angiogenesis.

Studies have pointed out [16] that the spiral arteries of damaged endometrium basal layer have rich blood flow, high oxygen tension, and increased production of reactive oxygen species (ROS), which could lead to sustained oxidative stress(OS). The oxidative stress microenvironment could reduce the survival rate and homing rate of exogenous transplanted MSCs. Based on the advantages of stem cell transplantation and modern biomaterials, thermo-sensitive EPL-HP hydrogel was prepared using poloxamer-407(poly(ethylene oxide) poly (propylene oxide) poly-(ethylene oxide) copolymer, PEO-PPO-PEO) and low molecular heparin as the basic raw materials and EPL as the functional excipient and further encapsulated with BMSCs to explore the potential mechanism of the therapeutic effect of new formulations on endometrial injury.

## 2 Results

### 2.1 Characterization and labeling of BMSCs

**Fig. 1:**
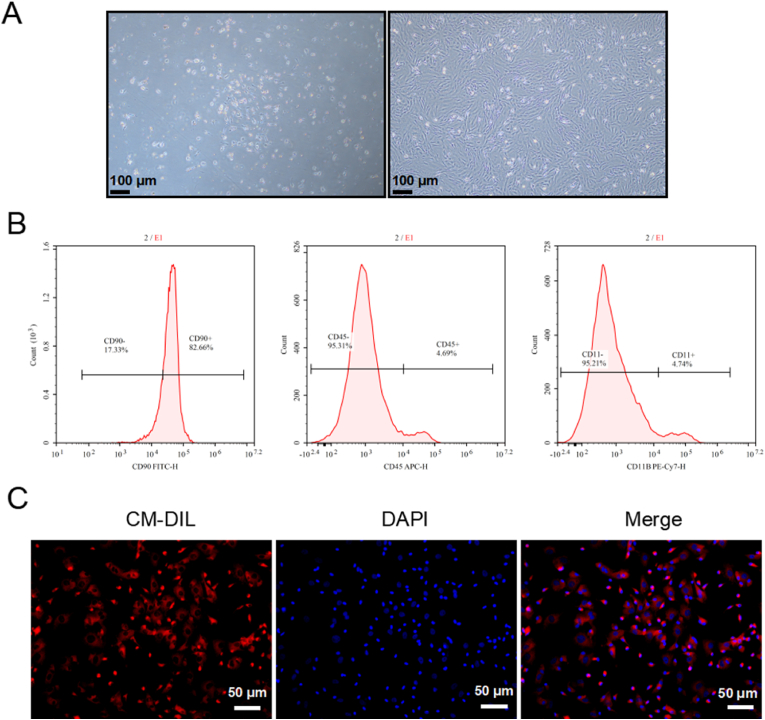
**Characterization, identification and labeling of BMSCs.** A. Primary BMSCs were cultured for 48 h and the third generation, Magnification: 100×, Scale bars = 100 μm; B. Identification of the third generation of BMSCs by flow cytometry, n = 3; C. CM-DIL labeled BMSCs in vitro, n = 3. Magnification: 200×, Scale bars = 50 μm.

Upon microscopic examination, the initially extracted primary cells from rats were found to be compact circular and suspended. After the cells were cultured for 48 h, most of the cells had various shapes, including round, spindle or irregular. On the 7th day of culture, the cells gradually merged into sheets and formed colonies, and were passaged when reaching 80 %–90 % confluence. The third generation BMSCs exhibited relatively homogeneous, fusiform and swirling shapes, as shown in Fig. 1-A. The flow cytometry identification results showed that the positive expression rate of CD90 was 82.66 %, the negative expression rate of CD45 was 95.31 %, and the negative expression rate of CD11b was 95.21 %, as shown in Fig. 1-B. Under a fluorescence microscope, the cytoplasm and membrane of BMSCs exhibited red fluorescence. The labeling rate could reach (99.8 ± 0.2)%, as shown in Fig. 1-C. Therefore, these results showed that the isolation, culture and labeling of BMSCs were successful.

### 2.2 Characterization of the thermo-sensitive EPL-HP hydrogel

**Fig. 2:**
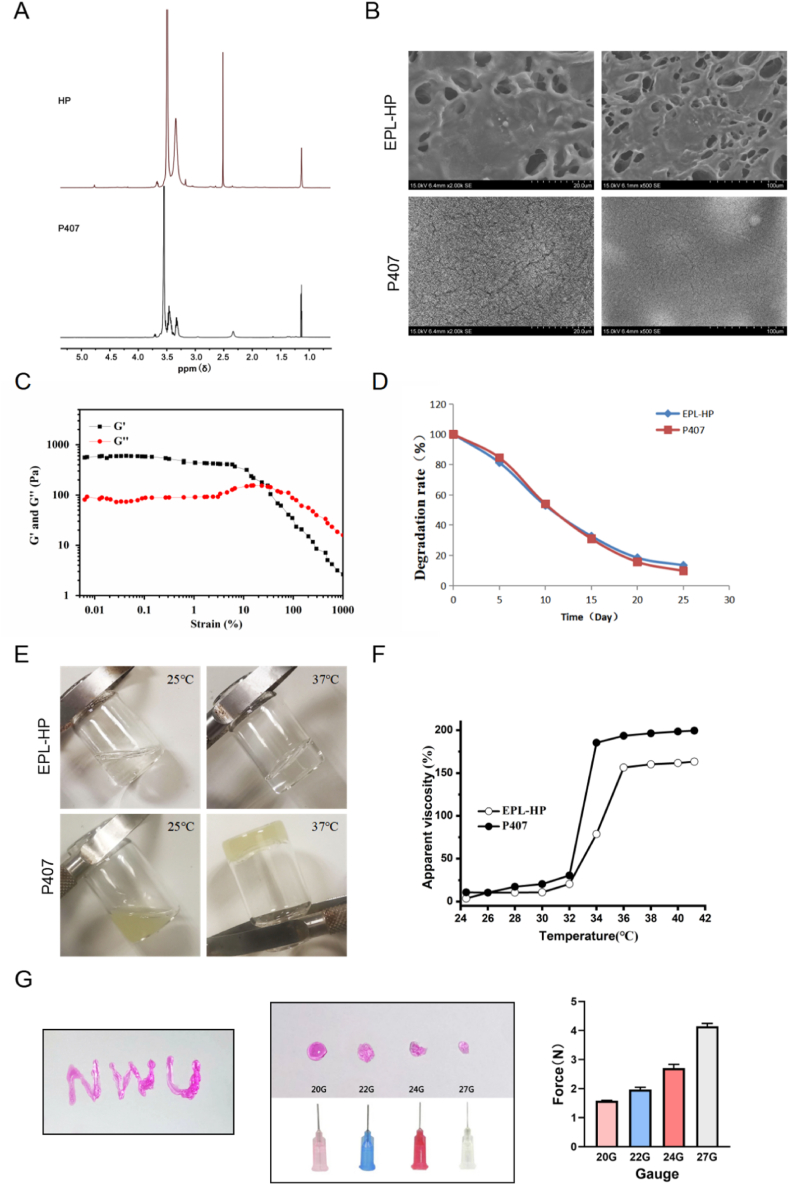
**Characterization of thermo-sensitive EPL-HP hydrogel.** A. Mass spectrometry analysis of EPL-HP hydrogel and P407 hydrogel; B. The morphology of two hydrogels were observed using SEM (20 μm, 100 μm); C. Strain sweep measurements of the storage moduli for EPL-HP hydrogel; D. Two hydrogels in vitro degradation rate curves; E. Gel morphology of two hydrogels; F. Viscosity-temperature curves of two hydrogels; G. Evaluation of injectability of EPL-HP hydrogel.

Mass spectrometry detection: P407:1.14, 3.4, and 3.5 ppm for–CH3,–CH,–CH_2_ in group of PPO, d = 3.65 ppm for–CH_2_ of PEO. HP: Combined with existing literature reports [17], there are aliphatic peaks of -CH-CO and -CH_2_-O of HP at 1.14 ppm and 3.65 ppm to confirm the binding of HP to the P407 main chain, as shown in Fig. 2-A. In this study, low molecular weight heparin and cationic peptide EPL were used to prepare a temperature-sensitive EPL-HP bioadhesive hydrogel. The EPL-HP hydrogel exhibits a porous three-dimensional network that can facilitate cell distribution, while the P407 hydrogel exhibits a non-porous rough plane when observed under a scanning electron microscope (SEM), as shown in Fig. 2-B. It was found that both elastic modulus G′ and viscous modulus G″ of the EPL-HP hydrogel decreased with increasing strain force, reaching almost identical at 30 % with a pressure of 150 Pa, as shown in Fig. 2-C.

The results of the hydrogel degradation rate curve (W/W0) showed that at 37 °C, the hydrogel could accumulate for 25 days, as shown in Fig. 2-D. As shown in Fig. 2-E, when the temperature rose to 37 °C, the EPL-HP hydrogel phase changed from sol state to stable gel state without any other precipitation, which was better than that of P407. Compared with the P407 hydrogel, the phase transition gel temperature and gelation time of EPL-HP hydrogel were significantly higher, as shown in Fig. 2-F and Table 2. With a 20 G size syringe, the minimum injection force required is achieved, up to 1.563 N, as shown in Fig. 2-G. Compared with P407 hydrogel, these results indicated that the temperature-sensitive EPL-HP bioadhesive hydrogel is more suitable to facilitate tissue repair at the injury sites as a drug delivery material for in vivo transplantation.

**Table 1:** Primer information for qRT-PCR.

Gene	Forward primer	Reverse primer
VEGF	5′-GCACAAGGACGGCTTG AAGAT-3′	5′-CCCACGGAGAAGA GCAGA-3′
SDF-1α	5′-TGCACAATGGAGCTTTTATAAC-3′	5′-AAAGCAAACCGAATACAGAC-3′
CXCR4	5′-AGGCCGTCTATGTGGGTGTCTGG-3′	5′-GAGGGCCTTGCGCTTCTGG-3′
HIF-1α	5′-CTACAAGAAACCGCCTATGACG-3′	5′-GGCTCATAACCCATCAACTCAG-3′
GAPDH	5′-CTGGAGAAACCTGCCAAGTATG-3′	5′-GGTGGAAGAATGGGAGTTGCT-3′

**Table 2:** Gel time and gel temperature of two hydrogels.

Group	EPL-HP hydrogel	P407 hydrogel
Concentration (%)	17 %	20 %
Gelation Time (sec)	45.6	29.8
Gelation Temperature (°C)	34.6	33.2

### 2.3 In vivo and in vitro evaluation of thermo-sensitive EPL-HP hydrogel-encapsulated BMSCs

**Fig. 3:**
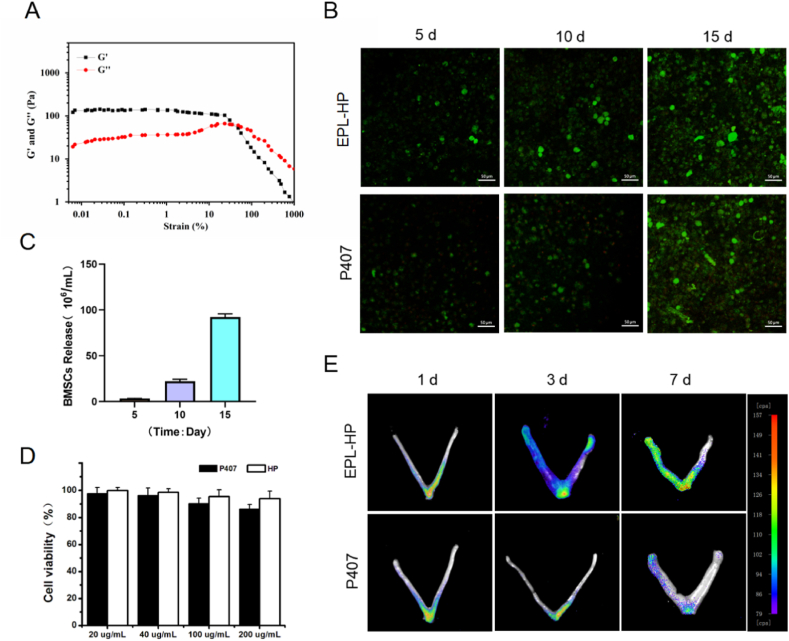
**The thermo-sensitive EPL-HP hydrogel-encapsulated BMSCs has good biocompatibility.** A. Strain sweep measurements of the storage moduli for EPL-HP hydrogel encapsulated BMSCs; B. Two hydrogels encapsulated BMSCs of dead/live cells, n = 3; C. Amount of BMSCs released by EPL-HP hydrogel; D. CCK-8 detects the biocompatibility of two hydrogels encapsulated BMSCs; E. Results of fluorescence distribution of two hydrogels encapsulated BMSCs in the uterine cavity (Time: 1 day, 3 day and 7 day).

At 35 % strain, G ′and G″ pressures were 62.5 Pa, indicating that encapsulated BMSCs had little effect on the mechanical properties of EPL-HP hydrogels, as shown in Fig. 3-A. Compared to P407 hydrogel, BMSCs cultured in EPL-HP hydrogel, had only small numbers of dead cells, and were significantly increased in P407 hydrogel from 10 d, as shown in Fig. 3-B. Compared to P407 hydrogel, BMSCs cultured in EPL-HP hydrogel had only small numbers of dead cells, and were significantly increased in P407 hydrogel from 10 d, as shown in Fig. 3-B. The average number of BMSCs released by EPL-HP hydrogel was determined by cell counter, 3.5 × 10^6^/mL at 5 d, 22.3 × 10^6^/mL at 10 d and continued to increase to 92.3 × 10^6^/mL at 15 d, as shown in Fig. 3-C.

The CCK-8 results showed that the thermo-sensitive EPL-HP hydrogel had good cytocompatibility with EPL at 100 μg/mL, as shown in Fig. 3-D. In addition, the results of small animal imager showed that after 1 d of administration, there was no difference in fluorescence intensity between the two hydrogel groups, and it was concentrated at the bottom of the Y-shaped uterus, as shown in Fig. 3-E. As the duration extended to 3 d, the fluorescence intensity of the EPL-HP hydrogel group increased significantly and shifted to the left uterus. When it came to 7 d, it was found that the fluorescence intensity of the EPL-HP hydrogel group was significantly higher than that of the P407 hydrogel group, which indicated that the EPL-HP hydrogel had a better release of BMSCs and was more suitable as scaffold material to encapsulate BMSCs than the P407 hydrogel.

These results indicated that EPL-HP hydrogels are more suitable for the survival and release of BMSCs compared to P407 hydrogel, which is an important influencing factor as a drug delivery system.

### 2.4 In vivo analysis of endometrial thickness and glandular proliferation with the thermo-sensitive EPL-HP hydrogel encapsulated BMSCs

**Fig. 4:**
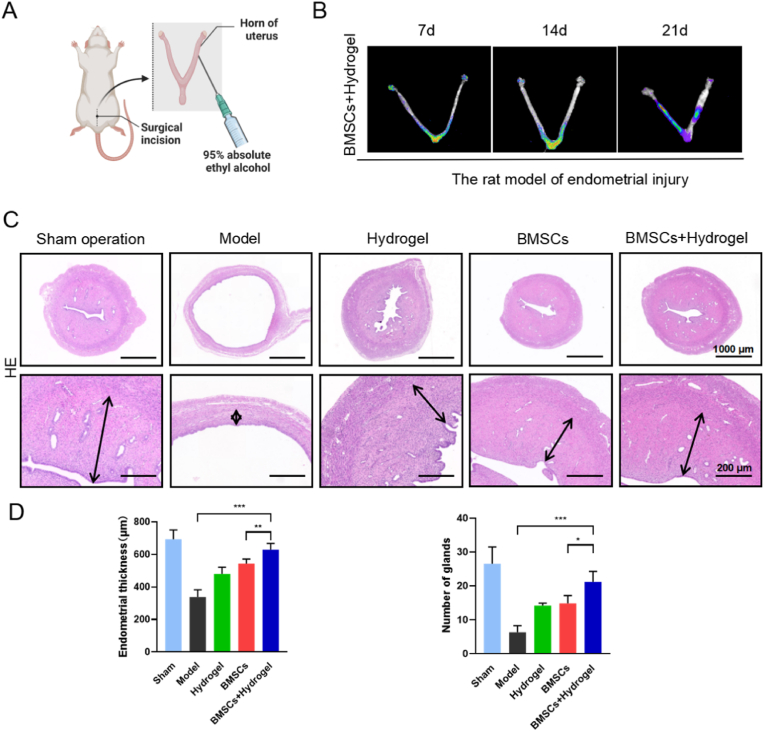
**The thermo-sensitive EPL-HP hydrogel encapsulated BMSCs could increase endometrial thickness and gland proliferation.** A. A simple schematic diagram of the rat model of endometrial injury established through intrauterine injection of 95 % absolute ethanol; B. Fluorescence intensity distribution diagram of EPL-HP hydrogel encapsulated BMSCs to repair the rat model of endometrial injury (Time: 7, 14 and 21days); C. H&E staining pictures of the rats' uteri in each group. Black arrows indicate endometrial thickness, scale bars = 1000 μm, 200 μm; D. Histogram of endometrial thickness and the number of glands of rats in each group, n = 6. Compared with the BMSCs + Hydrogel group, ∗*P* < 0.05, ∗∗*P* < 0.01, ∗∗∗*P* < 0.001.

In order to determine the optimal intervention time for EPL-HP hydrogel encapsulated BMSCs to repair the rat model of endometrial injury, we collected the uteri to observe the fluorescence intensity on the days 7, 14 and 21, as shown in Fig. 4-B. The results showed that the fluorescence intensity was mostly concentrated near the bottom of the Y-shape on the days 7 and 14, while the fluorescence intensity was mainly concentrated on the injury site on on the days 21. These results indicated that the thermo-sensitive EPL-HP hydrogel encapsulated BMSCs can better repair injured endometrium after 21 days of intervention.

The results of H&E staining showed that in the sham operation group, the endometrial structure of the rats remained intact, with tightly arranged epithelial cells exhibiting a single-layer columnar shape, and the interstitial cells were densely distributed. In contrast, in the model group, a significant reduction in the thickness of the rat's endometrium was observed, accompanied by disordered arrangement of epithelial and stromal cells, as well as infiltration of inflammatory cells, as shown in Fig. 4-C. These results indicated that the rat model of endometrial injury was successfully established using the 95 % ethanol chemical injury method, as shown in Fig. 4-A. Compared with the endometrial thickness of (338 ± 80) μm in the model group and that of (543 ± 38) μm in the BMSCs group, the endometrial thickness could increase to (630 ± 54) μm in the BMSCs + Hydrogel group, accompanied by well-distributed cells and obvious angiogenesis, as shown in Fig. 4-D. Compared with the (6 ± 2) glands in the model group and (15 ± 2) glands in the BMSCs group, the number of endometrial glands in the BMSCs + Hydrogel group could increase to (20 ± 3), as shown in Fig. 4-D. These results provided evidence for the potential of the thermo-sensitive hydrogel formulations in facilitating endometrial regeneration.

### 2.5 Effects of the thermo-sensitive EPL-HP hydrogel encapsulated BMSCs on apoptosis and inflammation in endometrial regeneration

**Fig. 5:**
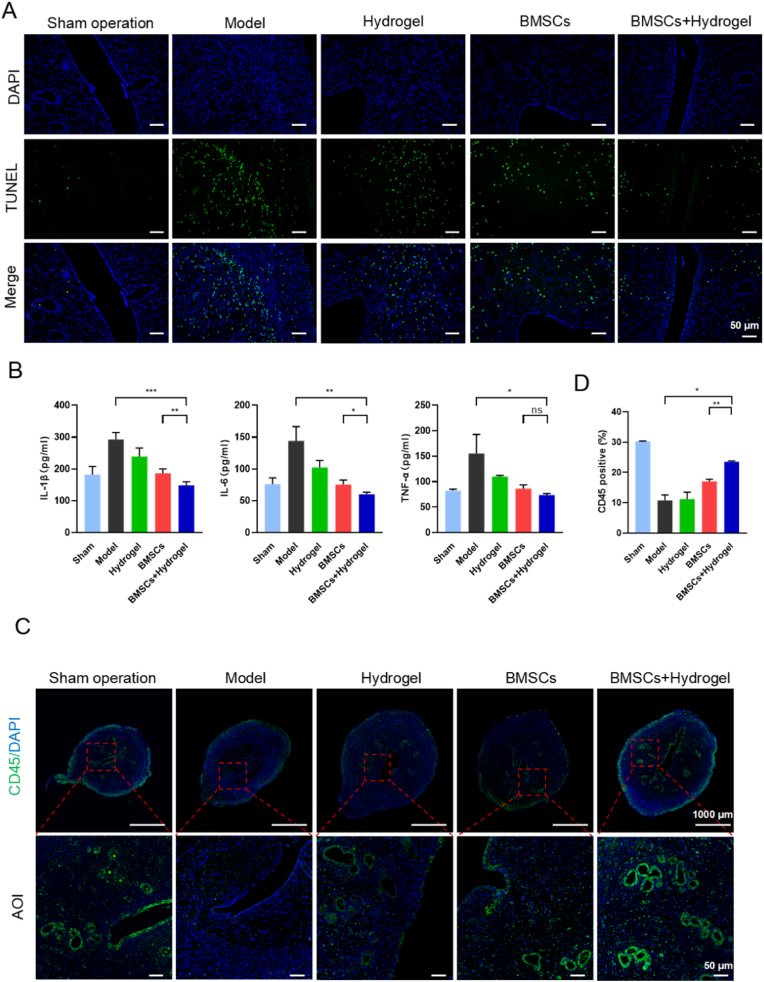
**The thermo-sensitive EPL-HP hydrogel encapsulated BMSCs can alleviate the effects of apoptosis and inflammation in endometrial repair.** A. TUNEL staining to evaluate endometrium cell apoptosis, n = 3, Scale bars = 50 μm; B. Histogram of IL-1β, IL-6 and TNF-α concentration in the uterus of each group was detected by ELISA, n = 6; C. Immunofluorescence staining for CD45 to observe immune cell infiltration, Scale bars = 50 μm; E. Histogram of CD45 fluorescence intensity expression in the uterus of each group, n = 3. Compared with the BMSCs + Hydrogel group, ∗*P* < 0.05, ∗∗*P* < 0.01, ∗∗∗*P* < 0.001.

In clinical practice, endometrial stromal cells release pro-inflammatory factors (IL-1β, IL-6 and TNF-α) due to repeated uterine operations, long-term use of contraceptive pills and ovulation induction drugs, which in turn could cause apoptosis. These pro-inflammatory factors disrupt vascular integration during endometrial repair and affect endometrial microenvironment. Endometrial damage caused by inflammation and apoptosis may lead to increased incidence of embryo implantation failure, premature birth, and miscarriage. Therefore, this study used TUNEL and ELISA assays to assess the cell apoptosis rate and inflammation in the endometrial tissues of each group. The results of TUNEL staining showed that the apoptosis in the rat model of endometrial injury was mainly concentrated in endometrial stromal cells, rather than the epithelial cell layer. As shown in Fig. 5-A and B, BMSCs + Hydrogel group exhibited a significant increase in cell apoptosis and anti-inflammation on the injured endometrium, as compared to the BMSCs group. Under physiological conditions, immune cells can clear endometrial debris, produce various growth factors, and promote the repair and regeneration of endometrial tissue. Previous studies [[18], [19], [20]]found that after endometrial injury by mechanical damage, during the early inflammatory stage, the infiltration of CD45 cells is definitely increased. But we found that CD45 immune cells are reduced in the disease state, consistent with the findings of the article of Lv et al. [21]. This may be related the modeling method and intervention time. The results of CD45 staining showed that the immune cell infiltration was mainly concentrated around the glands and epithelium. As shown in Fig. 5-C, BMSCs + Hydrogel group exhibited a significant increase in immune cell infiltration, which promoted the regeneration of endometrium.

### 2.6 Evaluation of in vivo angiogenic and endometrial receptivity of the thermo-sensitive EPL-HP hydrogel encapsulated BMSCs

**Fig. 6:**
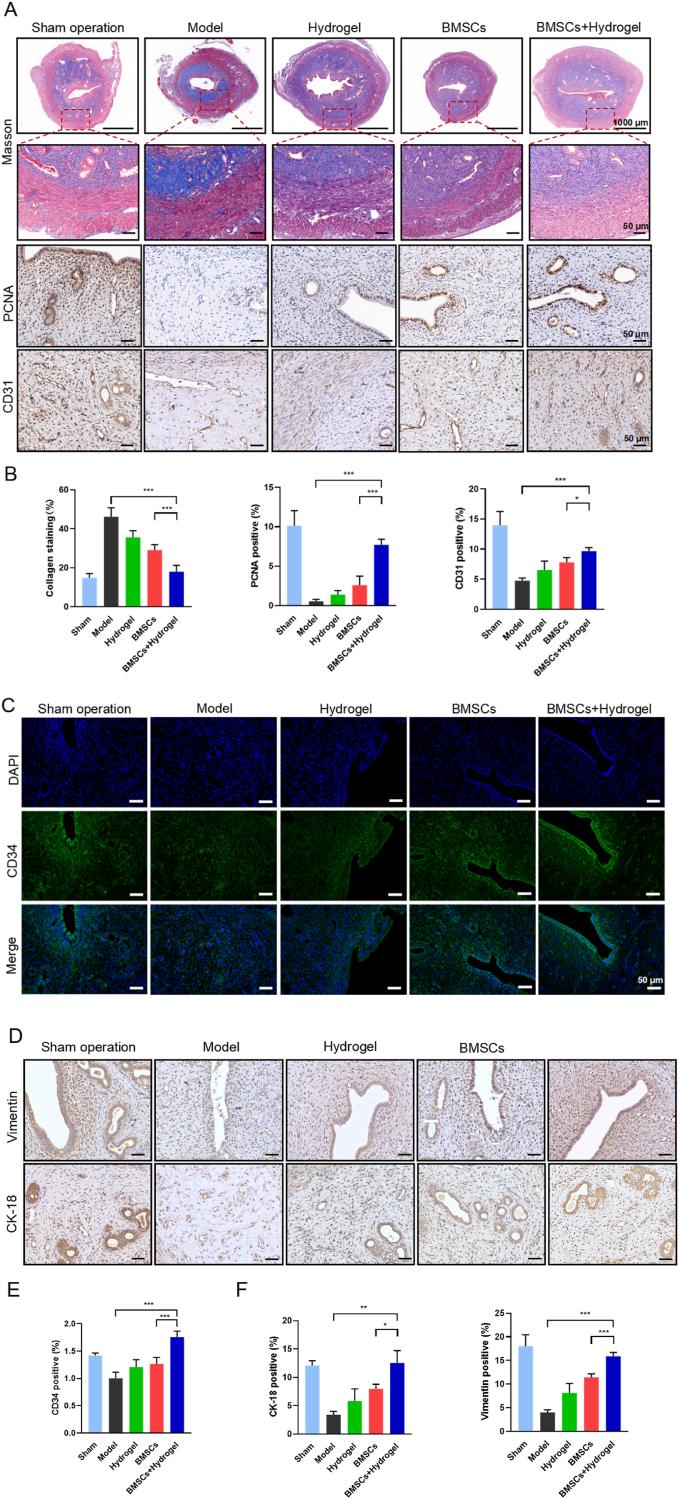
**The thermo-sensitive EPL-HP hydrogel encapsulated BMSCs could reduce endometrial collagen deposition, promote endometrial proliferation angiogenesis and endometrial receptivity in vivo.** A. Masson staining was used to evaluate the degree of endometrium fibrosis in each group, immunohistochemical staining PCNA was used to evaluate the endometrial proliferation in each group, and CD31 was used to evaluate the angiogenesis of the endometrium. Scale bars = 1000 μm, 50 μm; B. Histogram of endometrial collagen deposition rate, expression rate of PCNA-positive cells and CD31-positive cells in the uterus of each group, n = 6; C. Immunofluorescence staining for CD34 to evaluate endometrium blood vessels. Scale bars = 50 μm; D. Immunohistochemical staining Vimentin was used to evaluate the production of endometrial stromal cells in each group, and staining CK-18 was used to evaluate the production of endometrial epithelial cells. Scale bars = 50 μm; E. Histogram of CD34 fluorescence intensity expression in the uterus of each group, n = 3; F. Histogram of the expression rate of Vimentin-positive cells and CK-18-positive cells of the uterus in each group, n = 6. Compared with the BMSCs + Hydrogel group, ∗*P* < 0.05, ∗∗*P* < 0.01, ∗∗∗*P* < 0.001.

Endometrial regeneration relies on cell proliferation and angiogenesis to better provide nutrition and oxygen to the regenerating tissue. Therefore, to assess the in vivo efficacy of the thermo-sensitive hydrogel formulations in promoting endometrial regeneration, we conducted analyses on the expression of endothelial cell marker CD31 and the cell proliferation factor PCNA. As shown in Fig. 6-A, the results showed that the BMSCs + Hydrogel group had the strongest staining effect, indicating that it had a more significant effect on promoting endometrial cell proliferation and angiogenesis. Masson's trichrome staining was used to evaluate the collagen deposition and fibrosis degree of the endometrium of rats in each group. The results showed that the endometrium of the model group had severe collagen fiber deposition. The muscle fibers are damaged, which could lead to myometrium weakness in severe cases and increase the rate of uterine rupture during pregnancy. The EPL-HP hydrogel encapsulated BMSCs could effectively alleviate endometrial inflammation and fibrosis by stabilizing and controlled-releasing BMSCs, promoting regeneration of damaged endometrium.

As the origin of interstitial cells, CD34 is a representative factor of microvessel density and provides nutritional support for endometrium regeneration [22]. As shown in Fig. 6-C, compared with the BMSCs group, the fluorescence expression intensity of the BMSCs + Hydrogel group was the strongest, which showed that it had the superior effect in promoting angiogenesis in the rat model of endometrial injury. Endometrium receptivity and angiogenesis are key factors for embryo implantation and maintenance of pregnancy. Vimentin is a marker protein for epithelial-to-mesenchymal transition (EMT) and stromal cells, and CK-18 is a marker protein for endometrial epithelial cells. Both are key indicators for evaluating endometrial receptivity. As shown in Fig. 6-D, compared with the model group and the BMSCs group, the BMSCs + Hydrogel group had the strongest staining, indicating that the new hydrogel formulations group can improve the receptivity of the rat model of endometrial injury. These results showed that the EPL-HP hydrogel encapsulated BMSCs could improve the controlled release rate and targeting efficiency of encapsulated BMSCs, thereby offering strong support for pregnancy functions.

### 2.7 Analysis of the mechanism of the thermo-sensitive EPL-HP hydrogel encapsulated BMSCs in repairing endometrial injury

**Fig. 7:**
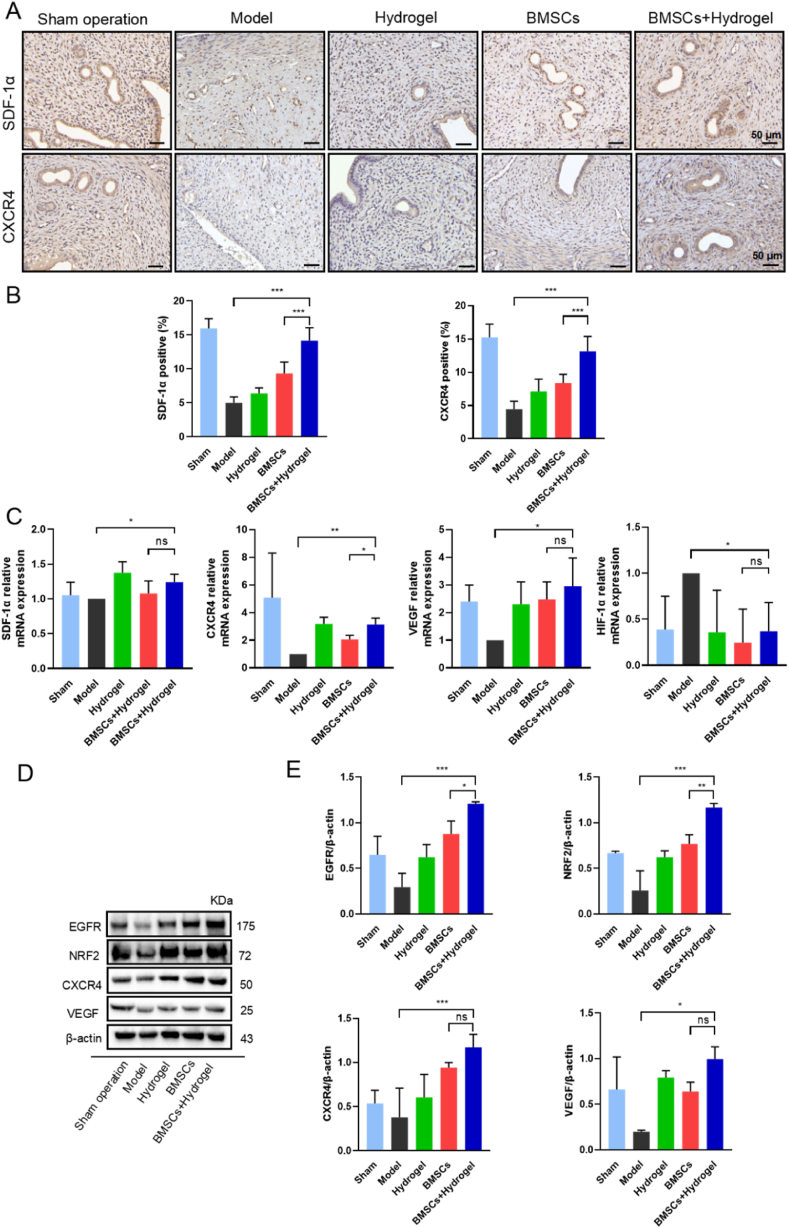
**The thermo-sensitive EPL-HP hydrogel encapsulated BMSCs regulate the SDF-1/CXCR4 axis by activating Nrf2 to repair damaged endometrium.** A. Immunohistochemical staining of SDF-1α and CXCR4 was used to evaluate the homing of endometrial BMSCs in each group. Scale bars = 50 μm; B. Histogram of the expression rate of SDF-1α and CXCR4 positive cells in the uterus of each group, n = 6; C. qRT-PCR detection of the expression of SDF-1α, CXCR4, VEGF and HIF-1α in the uterus of each group, GAPDH was used as a standardization control, n = 6; D. WB detected the protein expression of oxidative stress-related factors (Nrf2), angiogenesis-related factors (EGFR, VEGF), CXCR4 and β-actin in the uterus of each group. β-actin was used as a control. E. Histogram of EGFR, Nrf2, CXCR4, VEGF and β-actin mRNA in the uterus of each group, n = 3. Compared with the BMSCs + Hydrogel group, ∗*P* < 0.05, ∗∗*P* < 0.01, ∗∗∗*P* < 0.001.

Studies have confirmed [23,24] that the interaction between stromal cell-derived factor-1 (SDF-1) produced at the site of injury and the CXC family cytokine receptor 4 (CXCR4) expressed on the surface of BMSCs plays a crucial role in the migration and homing of exogenous BMSCs. Antioxidant stress factors Nrf2 is not only an effective target to improve the survival rate and homing rate of MSCs at the injury site, but also directly improve the blood supply and promote the self-repair function of endogenous cells. As shown in Fig. 7-D, the BMSCs + Hydrogel group could activate the protein expression of Nrf2 and CXCR4. Compared with the BMSCs group, the BMSCs + Hydrogel group could better mediate the release of BMSCs, more effectively activate the SDF-1/CXCR4 axis, and recruit more BMSCs to promote the repair of endometrial damage, as shown in Fig. 7-A. Nrf2 has been confirmed to directly bind to the promoter (CXCR4), stimulating the upregulation of the expression level of the surface receptor CXCR4 on MSCs, thereby activating the SDF-1/CXCR4 axis and promoting the migration and homing of exogenous MSCs to the site of injury [[25], [26], [27]]. From a genetic perspective, the thermo-sensitive EPL-HP hydrogel encapsulated BMSCs can activate the SDF-1/CXCR4 biological axis and promote the expression of the vascular endothelial growth factor (VEGF), which is also consistent with the results of immunohistochemical staining, as shown in Fig. 7-C.

Studies [28] have reported that in certain disease processes, overexpression of epithelial endothelial growth factor(EGFR) could stimulate the expression of VEGF, promote cell proliferation and the expression of related angiogenic factors. This study found that the expression of angiogenesis-related factors EGFR and VEGF decreased during the development of endometrial injury, and the BMSCs + Hydrogel group could increase the protein level expression of angiogenesis-related factors. This finding indicated that the thermo-sensitive EPL-HP hydrogel encapsulated BMSCs could activate angiogenesis and restore blood supply to the injured endometrium, which is beneficial to embryo implantation and maintenance of pregnancy.

HIF-1α is an indicator of oxygen homeostasis and is key for cells in menstrual and secretory phase to adapt to hypoxia. The production of VEGF under hypoxia is regulated by HIF-1α [29]. Animal experiments confirmed the negative correlation between the expression of HIF-1α and VEGF. The occurrence of endometrial injury is related to insufficient endometrial hyperplasia, hypoxia and significant reduction in endometrial angiogenesis [30]. The above results showed that the thermo-sensitive EPL-HP hydrogel encapsulated BMSCs could regulate the SDF-1/CXCR4 biological axis by activating Nrf2, exerting anti-apoptotic, anti-inflammatory and pro-angiogenic effects to regulate the endometrial microenvironment.

### 2.8 Evaluation of the ability of the thermo-sensitive EPL-HP hydrogel encapsulated BMSCs to promote endometrial regeneration in vivo

**Fig. 8:**
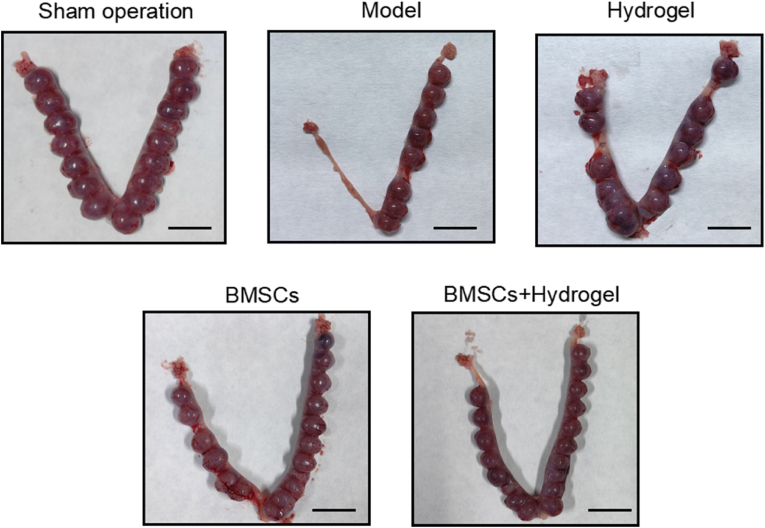
**The thermo-sensitive EPL-HP hydrogel encapsulated BMSCs can effectively promote endometrial regeneration in vivo.** The picture of the number of embryos conceived in the uterus in each group. Figures show injury was only induced to one side of the uterus.

In order to evaluate the recovery of pregnancy function in rats with endometrial injury by *the thermo-sensitive EPL-HP hydrogel encapsulated BMSCs*, we collected the number of pregnant rats, average conception time and average number of embryos in each group. After confirming rats’ pregnancy by vaginal smear, the rats uteri were taken out on the 13.5th day of pregnancy and the number of embryos was counted. As shown in Table 3, the number of pregnant rats in the hydrogel group and the BMSCs group were the same, while the number of pregnant rats in the BMSCs + Hydrogel group was the highest. This showed that the thermo-sensitive EPL-HP hydrogel encapsulated BMSCs could better promote fertility recovery and endometrial regeneration. From the perspective of embryo quality and conception time, the BMSCs + Hydrogel group was significantly better than the Hydrogel group, which showed that the thermo-sensitive EPL-HP hydrogel encapsulated BMSCs could achieve effective controlled release of BMSCs, thereby improving the implantation rate and embryo survival rate, as shown in Fig. 8 and Table 3. The prepared thermo-sensitive EPL-HP hydrogel encapsulated-BMSCs has the characteristics of high tissue affinity, high adhesion and controlled release. Consequently, these characteristics offer a promising therapeutic option for endometrial regeneration, ensuring clinical safety.

**Table 3:** Number of pregnant rats, average time to conception, and average number of embryos in each group(n = 5).

Variable	Sham	Model	Hydrogel	BMSCs	BMSCs + Hydrogel
Numbers of pregnant	5	0	1	1	2
Time of conception	2	4	3.5	3	1.5
Numbers of embryos (mean ± SEM)	7.2	0	1.2	1.2	2.8

## 3 Discussion and conclusion

Endometrial injury is often caused by various improper uterine cavity operations or postoperative infections, which could damage the basal layer and blood supply of the uterine cavity, causing inflammatory degeneration. It may lead to regeneration disorders of stromal cells and endometrial epithelial cells, decrease the number of endometrial glands and endometrial receptivity, affect the implantation of fertilized eggs or embryos, and even cause female infertility or recurrent miscarriage. Previous studies [31] have discovered the feasibility of MSCs differentiation into endometrial cells, providing experimental basis for the application of stem cell therapy after endometrial injury.

In this study, in order to improve the survival rate and homing rate of BMSCs, we planned to first modify the poloxamer hydrogel and graft the negative linear polysaccharide low molecular weight heparin poloxamer (HP) to prepare HP hydrogel. Heparin has affinity with various cell growth factors and gathers the cell growth factors in surrounding tissues inside the material and the endometrium. Secondly, based on the characteristic that stem cell membranes, HP hydrogel and endometrial mucus are all negatively charged, the cationic peptide EPLwas further introduced to enhance the binding strength between BMSCs, HP hydrogel and endometrium. Through electrostatic interaction, a thermo-sensitive EPL-HP hydrogel is prepared and it is expected to ensure adhesion of BMSCs within the biological scaffold, combined with the controlled-release effect of EPL-HP hydrogel to promote endometrial regeneration in the long term.

Previous studies found that EPL-HP hydrogel could promote endometrial regeneration. In this study, compared with P407 hydrogel, we further found that the thermo-sensitive EPL-HP bioadhesive hydrogel has a porous three-dimensional network, good biocompatibility, good degradability and good injectability. In vivo imaging experiments further demonstrated that EPL-HP hydrogel had better BMSCs encapsulation effect and was suitable to exert the potential of promoting endometrial regeneration. It could improve the low adhesion of biomaterials-tissue in the injury site, low survival rate and homing rate of transplanted BMSCs. It could increase the combining strength between BMSCs-biomaterials-tissue, and then improve the therapeutic effect of BMSCs transplantation on the rat model of endometrial injury.

However, according to the current research results [32], due to the continued increase in oxidative stress levels at the endometrial injury site and the oxidative stress microenvironment, the survival rate and homing rate of exogenously transplanted MSCs are low. Therefore, the therapeutic effect still needs to be further improved, and we will explore targeted solutions based on the regulation of relevant signaling mechanisms. Research has confirmed that the anti-oxidative stress transcription factor Nrf2 is a key regulator of MSCs stemness maintenance, survival, proliferation, differentiation and migration. It not only improves the survival rate of MSCs at the site of injury, but is also an effective target for inducing the homing, proliferation and differentiation into endometrial cells [33]. Nrf2 is activated in response to endometrial injury sites, maintains redox homeostasis within MSCs, and protects MSCs from cell death triggered by hypoxia and oxidative stress [34]. SDF-1 accelerates blood supply remodeling, mediates the recruitment of CXCR4+ hematopoietic cells, and upregulates the expression of VEGF, thereby synergistically promoting angiogenesis at the injury site [35].

**Fig. 9:**
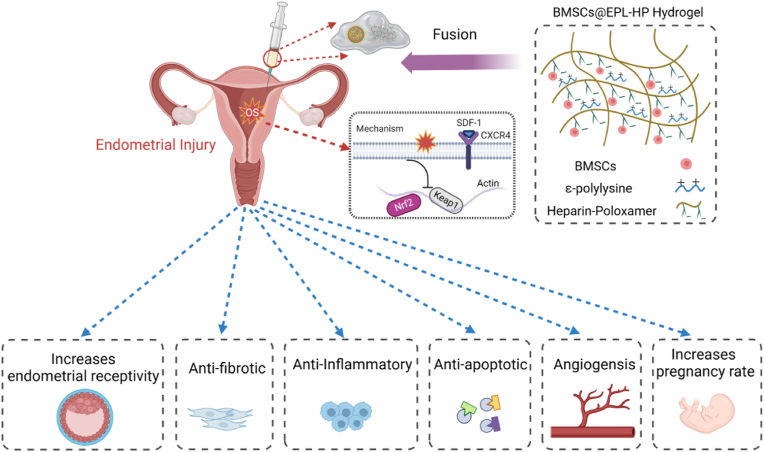
Simple flow chart of the article.

The thermo-sensitive EPL-HP hydrogel-encapsulated BMSCs was implanted into the uterine cavity of the rat model of endometrial injury, and it was found that it could regulate the SDF-1/CXCR4 biological axis by activating Nrf2 to promote endometrial angiogenesis, anti-fibrosis, anti-inflammation, anti-apoptosis and other aspects, which improved endometrial receptivity, promoted endometrial regeneration, and restored the pregnancy function of rats with damaged endometrium, as shown in Fig. 9.

The thermo-sensitive EPL-HP hydrogel encapsulated BMSCs has high tissue affinity, high viscoelasticity and controlled release of BMSCs, which could promote better homing of BMSCs after transplantation into the rat endometrial injury model to repair damaged endometrium. This study combines the advantages of stem cell transplantation and biological materials to develop a new formulation with broad application prospects and transformational value, and can provide a new solution for clinical treatment of endometrium injury. From all the embryo images provided, the difference of pregnancy rate between the Hydrogel combined with BMSCs group and BMSCs-only group is not very obvious. This may be related to the insufficient sample size for fertility testing in this study, which we need to pay attention to the number of sample in the future research.

## 4 Experiment

### 4.1 Isolation, culture, identification and fluorescent labeling of BMSCs

#### 4.1.1 Isolation and culture of BMSCs

3-week-old SPF female SD rats were sacrificed by cervical dislocation, and density centrifugation method [36] was used to extract BMSCs. Briefly, the cell suspension was transferred to a 15 mL centrifuge tube containing lymphocyte separation solution and centrifuged at 1000 rpm for 5 min to obtain the middle clear layer as the 0 generation. It was then placed in DMEM/F12 (Gibco) with 10 % FBS (Gibco) and cultured in a constant temperature incubator at 37 °C with 5 % CO_2_.

#### 4.1.2 Identification of BMSCs

The third generation of BMSCs were also used for FACS analysis. The cell suspension was centrifuged at 1000 rpm for 5 min, and the cell concentration of each flow tube was adjusted to approximately 10^6^/mL. 5uL of the following antibodies (CD90 FITC, ebioscience; CD45 APC, ebioscience; CD11b PE-Cy7, Biolegend) were added to the suspension, and then incubated at room temperature for 1 h. The cells were resuspended in 200 μL PBS, and flow cytometry was used to detect antigen expression of cell surface.

#### 4.1.3 CM-DIL labels BMSCs

The third generation of BMSCs with an adhesion rate of 80∼90 % were taken. The cell concentration was adjusted to approximately 6x10^5^/mL per well. 300 μL of prepared labeling solution (CM-DIL (Invitrogen): DMEM/F12 = 1:200) was added to each well after adhesion, and incubated in the incubator for 20 min. It was fixed with 4 % paraformaldehyde (Biosharp) for 10 min, and added with 300 μL of DAPI (Beyotime) for 5 min at room temperature. After washing twice with PBS, we observed the labeling situation under a fluorescence inverted microscope and took pictures. The labeling rate was calculated as (CM-DIL Co-labeled cells with DAPI/DAPI-labeled cells) × 100 %.

### 4.2 Preparation and characterization of EPL-HP hydrogel

#### 4.2.1 HP polymer synthesis and NMR detection

Step 1: 1 mM P407 (Sigma) and 1.3 mM 4-nitrophenyl chloroformate (Shanghai Ronen Reagent Co., Ltd) were dissolved in 20 mL of methylene chloride (Sinopharm Chemical Reagent Shanghai Co., LTD) (containing a slight excess of triethylamine (Sinopharm Chemical Reagent Shanghai Co., LTD)) at room temperature for 4 h. Subsequently, the resulting mixture was subjected to extraction with petroleum ether, a process that was repeated three times, to obtain derivatized 4-nitrophenylcarboxylate intermediate. Step 2: 1 mM intermediate and 3 mM diaminoethylene were dissolved in 20 mL dichloromethane (Sinopharm Chemical Reagent Shanghai Co., LTD) at room temperature and stirred overnight. After the reaction, it was extracted with petroleum ether for three times, then dialyzed with distilled water for 3 days to obtain the product amination P407 through lyophilization. In addition, the carboxyl group of heparin salt (2.5g, 0.5 mM, Aladdin reagent) was activated with EDC (0.106g, 0.5 mM, Shanghai Ronen Reagent Co., Ltd)/NHS (0.032g, 0.25 mM, Shanghai Ronen Reagent Co., Ltd). The final step: The amine group of poloxamer 407 and the carboxylic group of heparin were coupled for 24 h at room temperature to form an amide bond. After the reaction, the mixture was dialyzed with distilled water through a dialysis bag (MWCO = 14000) for 3 days, so as to obtain the product through lyophilization.

#### 4.2.2 Preparation of EPL-HP bioadhesive hydrogel

1 g of HP lyophilized powder and P407 were collected, and added a certain volume of distilled water until the mass fractions were 17 % of HP and 20 % of P407 of the hydrogel. After complete dissolution, an appropriate amount of EPL powder (20–200 μg/mL) was added to the HP hydrogel to prepare an EPL-HP hydrogel.

#### 4.2.3 Quality evaluation of EPL-HP bioadhesive hydrogel

*①Microscopic morphology observation:* An appropriate amount of EPL-HP hydrogel and P407 hydrogel samples were taken and quickly frozen to a solid state with liquid nitrogen, and placed in a freeze dryer for 48 h. The obtained sample was in the shape of a white block. After vacuum spraying with gold, its three-dimensional structure and surface micromorphology were observed using SEM.

*② In vitro degradation rate curve:* 0.4g of EPL-HP and P407 were prepared into a hydrogel of corresponding concentrations, and were quickly introduced to the dialysis tube (dialysis molecular weight 5000) in a low-temperature solution state. Subsequently, the dialysis tube was immersed into the dissolution tank containing 1 L of PBS solution, the stainless steel cage was rotated at a speed of 100 rpm at 37 °C, and the timer was started at 0. The steel cage was taken out at the set time points (0, 5, 10, 15, 20, 25 days) and placed under freeze-dryer. After lyophilization, the hydrogel mass(W) was accurately measured and the hydrogel degradation rate curve was drawn (W/W0).

*③Phase transition temperature determination:* The apparent viscosity of EPL-HP hydrogel was determined using coaxial cylinder rheometer. At the temperature of 24∼42 °C, the viscosity values of EPL-HP hydrogel and P407 hydrogel were recorded under corresponding conditions, and the viscosity-temperature curve was drawn.

*④Detection of Rheological Properties:* Add 500 μL of EPL-HP hydrogel stock solution or EPL-HP hydrogel and BMSCs (5 × 10^5^/mL) suspension into the test disk of the rotary rheometer (Haake Mars 40, Germany), set the conditions at 37 °C and 1 Hz, and the oscillating strain range is 0.1 %–1000 %, lasting for two cycles. The mechanical properties of EPL-HP hydrogel and the effect of loading BMSCs on the mechanical properties of EPL-HP hydrogel were evaluated with the elastic modulus G ′and the viscous modulus G' ‘as the test indicators.

*⑤Evaluation of injectability of EPL-HP hydrogel:* The morphological adaptability of EPL-HP hydrogel was tested by drawing the word “NWU” from the needle tube. In addition, in the compression mode, the shape and injection force of different specifications (20, 22, 24 and 27G) syringes were tested by the dynamic injection flow and pressure tester (SUMS PRING) at a rate of 0.35 mm/s, so as to determine the injection force of EPL-HP hydrogel (n = 3). The maximum force during the experiment is considered as the injection force, and the image is captured by a camera under natural light.

### 4.3 In vivo and in vitro evaluation of the novel thermo-sensitive EPL-HP bioadhesive hydrogel encapsulated BMSCs

#### 4.3.1 Living/dead cell experiment of EPL-HP hydrogel loaded with BMSCs

Mix BMSCs(5 × 10^5^/mL)with 1 mL EPL-HP hydrogel or P407 hydrogel stock solution. Under ultraviolet irradiation, the EPL-HP hydrogel/P407 hydrogel encapsulated BMSCs was formed within 30 s. Cells were cultured in hydrogel for 5 days, 10 days and 15 days, and the culture medium was changed every day. After staining with the Invitrogen kit for detecting live/dead cells, fluorescence images were taken under a confocal microscope (Nikon C1 Plus, Japan).

#### 4.3.2 Release behavior of EPL-HP hydrogel to BMSCs

BMSCs (5 × 10^5^/mL) and 1 mL EPL-HP hydrogel were mixed evenly, and 1 mL cell culture medium (containing 10 % FBS, Gibco) was added after UV crosslinking and curing at 37 °C, and the time was set as 0. On the 5th, 10th, and 15th day, blow and aspirate 0.1 mL of the upper layer of cell culture medium (supplemented with new culture medium), and count the number of BMSCs in the supernatant using a cell counter (n = 3).

#### 4.3.3 CCK8 detection of the effect of EPL-HP hydrogel on the activity of BMSCs in vitro

We inoculated the third generation of BMSCs into a 96-well plate at 1x10^4^ cells/well, and cultured them in cell incubator overnight. Following the aspiration of the culture medium, we added 100 μL of HP hydrogel or P407 hydrogel to each cell well. The concentration of EPL in the HP hydrogel was adjusted to 20, 40, 100, and 200 μg/mL respectively. Subsequently, the 96-well plate was placed in a cell incubator for 24 h, following which the hydrogel was rinsed and the cell culture medium was replaced. The CCK-8 reagent solution was added, and the absorbance at 450 nm was measured with microplate reader to determine cell survival rate.

#### 4.3.4 In vivo detection of the in situ adhesion ability of EPL-HP hydrogel encapsulated BMSCs

SPF female SD rats, aged 8 to 10-week-old and weighing (200 ± 20) g, were randomly divided into two groups: EPL-HP hydrogel group and P407 hydrogel group (n = 6). BMSCs (1x10^7^/ml) were mixed with the EPL-HP hydrogel solution or P407 hydrogel solution at 4 °C. Subsequently, 30 μL of the cell-hydrogel complex was transplanted into the rat uterine cavity using a syringe. After 1, 3, and 7 days, the uteri of 2 rats from each group were collected, and the surface of the uteri was rinsed with PBS. A small animal imager was used to compare the fluorescence distribution of EPL-HP hydrogel and P407 hydrogel in the rats’ uteri at different times to evaluate its in situ adhesion ability on the endometrium.

### 4.4 Model establishment and administration

In this study, SPF female SD rats, aged 8–10 weeks and weighing (200 ± 20)g, were utilized. Additionally, the rats were confirmed to have regular estrous cycles through the examination of vaginal smears. They were all purchased from the Guangdong Provincial Medical Experimental Animal Center (Certificate of Conformity: SYXK (Guangdong) 2018-0001), and were kept at a temperature of (22 ± 1)°C and a relative humidity of about 55 %. They have been kept on a 12-h day/night cycle, and are free to take in sufficient food and water in accordance with the 3R principle. The process had been reviewed by the Animal Ethics Committee of Guangzhou University of Traditional Chinese Medicine (Approval number: 20220905001).

After 7 days of routine feeding, referring to the early modeling method [37], a rat model of endometrial injury was successfully established with intrauterine injection of 95 % absolute ethanol on the day of estrus. Sham operation group: simple abdominal incision without uterine modeling; Model group: the proximal and distal portions of both sides of the uterine horn were gently clamped with two vascular clamps, intrauterine injection of 95 % absolute ethanol for 5 min. After removing vascular clamps, PBS thrice was given to further remove remaining ethanol; Animals in the experimental group then underwent reoperation after model development, with one end of the uterus being clipped. Hydrogel group: a syringe was used to inject 30 μL of EPL-HP hydrogel solution into the uterine cavity; BMSCs group: a syringe was used to inject 30 μL of BMSCs cell suspension (1x10^7^ cells) into the uterine cavity; BMSCs + Hydrogel group: a syringe was used to inject 30 μL of EPL-HP hydrogel encapsulated BMSCs with a total number of cells of 1x10^7^ cells into the uterine cavity. In the fourth estrous cycle after transplantation, on the day of estrus, 6 rats in each group were sacrificed by cervical dislocation. The uterine tissues were fixed with 4 % paraformaldehyde or collected at −80 °C for later indexes detection.

### 4.5 Histological and immunohistochemical staining experiments

After the uterine tissues of the rats were fixed with paraformaldehyde for 48 h, they underwent standard procedures of dehydration and embedding into sections. H&E staining was performed to evaluate changes in endometrial thickness and gland number in each group of rats. Observe one slice of each rat and randomly select four 200 × fields to observe the thickness of the endometrium (average count). The evaluation of endometrial fibrosis in each group of rats was conducted in accordance with the guidelines provided by Masson's trichrome staining kit (Solarbio). Immunohistochemical staining for CD31 (1:100, Abclonal) and PCNA (1:100, Santa Cruz) was used to evaluate endometrial angiogenesis and cell proliferation. Immunohistochemical staining for Vimentin (1:100, Abclonal) and CK18 (1:1000, Proteintech) was used to evaluate the receptivity and repair degree of the endometrium. Immunohistochemical staining for SDF-1α (1:200, abcam) and CXCR4 (1:200, abcam) was used to evaluate the recruitment, survival rate and homing rate of BMSCs in the endometrium. All slides were scanned with 3D Histech Pannoramic MIDI II scanner and analyzed with CaseViewer software.

### 4.6 ELISA experiments

Weigh 10 mg of the fresh uterine tissues of the rats, and then grind it with a 9-fold homogenate medium. After centrifuging the grinding solution at 3000 rpm for 10 min, and taking the supernatant to prepare a 10 % tissue homogenate, the expression levels of inflammation-related factors (TNF-α, IL-1β, IL-6) were assessed according to the instructions of the ELISA kit (Jinglaibio, Shanghai). After assessing the absorbance at 450 nm using a microplate reader, a standard curve equation and sample concentration were obtained to evaluate the anti-inflammatory effect of the new formulations on endometrial injury.

### 4.7 TUNEL and immunofluorescence staining experiments

After fresh rat uterine tissues were embedded and sectioned with OCT, standard operations were performed according to the TUNEL apoptosis detection kit (Elabscience) to evaluate the improvement of the new formulations on the apoptosis of endometrial damaged epithelial cells and stromal cells. Immunofluorescence staining was performed to evaluate the recovery of endometrial microvessels. Briefly, sections were permeabilized with 0.5 % TritonX-100, blocked with 5 % donkey serum, incubated with primary antibody CD34 (1:200, Invitrogen) and CD45 (1:100, Invitrogen) overnight at 4 °C, and then incubated with secondary antibody donkey anti-rabbit (Alexa Flour 488, Affinity) at room temperature for1 hour. The slides were photographed with an inverted fluorescence microscope.

### 4.8 Quantitative real-time PCR (qRT-PCR)

First, NucleoZOL RNA extraction reagent was used to extract total RNA from uterine tissues. The cDNA was then synthesize by reverse transcription using HiScript®IIQ SelectRT SuperMix (Vazyme, Nanjing). Finally, cDNA was combined with SYBR using primer (see Table 1 for specific primer information), and added in sequence to the 96-well PCR reaction plate. GAPDH was used as an internal reference gene to compare the expression of relative genes and calculate the 2^−ΔΔct^ value.

### 4.9 Western blotting (WB) experiments

RIPA lysis buffer supplemented with PMSF (1:100) was employed to lyse the uterine tissues, followed by centrifugation to obtain the supernatant using low-temperature tissue grinder (Servicebio). The concentration of the supernatant was measured and balanced using the BCA protein concentration determination kit (Beyotime). It was transferred to a PVDF membrane by electrophoresis, followed by blocking and incubation with the following primary antibodies overnight (EGFR, 1:5000, Abmart; NRF2, 1:1000, Proteintech; VEGF, 1:1000, Abcam; CXCR4, 1:1000, Abcam; β-actin, 1:5000, Abcam). The corresponding secondary antibodies (Goat anti-rabbit IgG (H + L), 1:5000, Affinity; Goat anti-mouse IgG (H + L), 1:10000, Affinity) were incubated at room temperature for 1 h. The strips were detected on a multifunctional imager (BIO-RAD) and analyzed using Image J software. β-actin was used as an internal reference for normalization control.

### 4.10 Fertility testing

In the fourth estrous cycle after transplantation, the SD female rats from each group (n = 5) and male rats were placed in a cage at a ratio of 1:1 for mating. The day when the vaginal plug was detected was designated as the 0.5th day of pregnancy, and the pregnant rats were sacrificed on the 13.5th day. The number of pregnant rats, the average conception time and the average number of embryos in each group were collected to reflect the recovery of pregnancy function in rats with endometrial injury after treatment.

## 5 Statistical analysis

All data were analyzed through one way ANOVA by SPSS Statistics 26.0. When the variances were homogeneous, pairwise comparisons were performed by LSD. When the variances were unequal, the Dunnett's T3 method was used for analysis. If *P* < 0.05, it is considered to be statistical significance.

## CRediT authorship contribution statement

**Ruifang Han:** Writing – original draft, Methodology, Investigation, Data curation. **Haiyi Zhou:** Methodology, Data curation. **Xingshan Liang:** Methodology, Data curation. **Siyi He:** Writing – review & editing. **Xiaoming Sun:** Writing – review & editing. **Yongge Guan:** Funding acquisition, Conceptualization. **Yang Song:** Project administration, Funding acquisition, Conceptualization.

## Disclosure

The author reports no conflicts of interest in this work.

## Declaration of competing interest

We declare that we have no financial and personal relationships with other people or organizations that can inappropriately influence our work, there is no professional or other personal interest of any nature or kind in any product, service and/or company that could be construed as influencing the position presented in, or the paper of the manuscript entitled.

## Data Availability

Data will be made available on request.
